# I Cannot Read Your Eye Expression: Suicide Attempters Have Difficulties in Interpreting Complex Social Emotions

**DOI:** 10.3389/fpsyt.2020.543889

**Published:** 2020-11-10

**Authors:** Inés Ferrer, Adrián Alacreu-Crespo, Alicia Salvador, Catherine Genty, Jonathan Dubois, Maude Sénèque, Philippe Courtet, Emilie Olié

**Affiliations:** ^1^Laboratory of Social Cognitive Neuroscience, Psychobiology-IDOCAL, Faculty of Psychology, University of Valencia, Valencia, Spain; ^2^PSNREC, Univ Montpellier, INSERM, CHU de Montpellier, Montpellier, France; ^3^Department of Emergency Psychiatry and Acute Care, Lapeyronie Hospital, CHU Montpellier, Montpellier, France; ^4^FondaMental Foundation, Créteil, France

**Keywords:** RMET, emotion recognition, suicide attempt, major depressive disorder, severity

## Abstract

**Background:** The ability to differentiate emotions in social contexts is important for dealing with challenging social situations. Suicide attempters show some difficulties in emotion recognition that may result in hypersensitivity to social stress. However, other studies on the recognition of social complex emotions found that suicide attempters have similar performances as depressed non-attempters.

**Objectives:** To investigate differences in social emotion recognition in patients with current Major Depressive Episode (MDE) with and without history of suicide attempt.

**Methods:** Two hundred and ten patients with MDE were recruited among whom 115 had lifetime history of suicide attempt (suicide attempters, SA) and 95 did not (affective controls, AC). Recognition of complex social emotions was assessed using the Reading the Mind in the Eyes Test (RMET). Emotions were separated in three valence categories: positive, negative, and neutral. Verbal intelligence quotient (IQ) and attention were measured with the National Adult Reading Task (NART) and the d2 test, respectively.

**Results:** Mixed logistic regression models adjusted for sex, lifetime bipolar disorder, verbal IQ and attention showed that the RMET performance for neutral emotions was worse in the SA than AC group (OR = 0.87 [0.75, 0.99]). Furthermore, when violent/serious SA were compared to non-violent/non-serious SA and AC, the RMET neutral valence category showed a trend for group factor (*p* < 0.059) and RMET scores were lower in the violent/serious SA than AC group (OR = 0.79 [0.64, 0.96]).

**Conclusion:** Recognition of neutral emotions is poor in SA and this may complicate their daily life. Interventions to improve the understanding of complex emotions may be helpful to prevent suicidal risk in patients with depression.

## Introduction

About 800,000 people commit suicide every year (1). People who complete or attempt suicide usually have psychiatric comorbidities, especially affective disorders, such as major depression (2). Suicide rate could be partially reduced by early detection of people at risk (2).

A recent meta-analysis in the framework of the Research Domain Criteria (RDoC) showed that disrupted social processes are a risk factor for suicide (3). Indeed, previous studies found that suicide attempters are more vulnerable to social stress (4). It is thus crucial to understand the mechanisms that make some individuals more vulnerable to suicidal acts in the presence of social adversity (5). Suicide attempters show decreased activation of the insula during a social exclusion paradigm (the Cyberball Game) compared with patients with history of depression without suicidal acts (6). Moreover, it has been hypothesized that deficits in social cognition are one of the mechanisms associated with hypersensitivity to social stress (4). Suicide attempters also show specific deficits in the interpretation of disgust, fearful (7) and angry faces (8). Some studies highlighted that emotion recognition depends on orbitofrontal cortex (9, 10). This region has been widely associated with suicidal vulnerability (11). Interestingly, suicide attempters showed an increased activation of the orbitofrontal cortex when viewing angry faces (8, 12). Difficulties in emotional recognition in suicidal patients may thus be related with a dysfunctional processing of emotional stimuli by orbitofrontal cortex.

Impairments in complex social emotion recognition can be evaluated using the Reading the Mind in the Eyes Test (RMET) (13). This widely used test can detect subtle impairment in positive, negative and neutral emotion recognition (14). A meta-analysis on RMET performance showed lower scores in patients with major depression and borderline personality disorder compared with healthy controls (15). However, studies in patients with suicide behavior are scarce. Patients with current major depressive episode (MDE), with and without history of suicide attempt, perform worse in all the RMET categories than healthy controls (16). RMET performance is worse in patients with depression and predominantly affective symptoms (including suicidal ideation) than in patients with depression characterized by predominance of somatic symptoms (17). Among elderly patients with current MDE and history or not of suicide attempts, the total RMET score was lower in lifetime suicide attempters than in healthy controls (18). The absence of differences between patients with depression with and without history of suicide behavior suggests that RMET performance impairment could be associated with a cognitive/affective dimension of MDE rather than with suicidal behavior. However, this lack of differences could also be explained by the small sample size of these studies and the different ages of the included patients.

The main aim of this study was to investigate whether social emotion recognition (RMET score) is impaired in suicide attempters compared with non-attempters in a sample of inpatients with MDE. We hypothesized that suicide attempters would perform poorly in recognizing negative emotions (7, 8). Moreover, we wanted to assess whether history of violent/serious suicide attempt or of repeated suicide attempts was associated with impaired RMET performance.

## Methods

### Participants

For this study, 210 patients (69% of women), aged between 18 and 70 year (mean ± SEM = 41.22 ± 0.89) were recruited at the Department of Emergency Psychiatry and Acute Care, Montpellier University Hospital, France. Patients were admitted for a current MDE, according to the DSM-IV criteria. Exclusion criteria were: current psychotic features, lifetime history of schizoaffective disorder or schizophrenia, euthymic status (i.e., Montgomery-Åsberg Depression Rating Scale, MADRS, score ≤ 7) (19), lifetime history of cerebrovascular accident and head trauma.

Among the 210 patients, 95 patients had no history of suicide attempt (affective controls, AC) and 115 had lifetime history of suicide attempt (suicide attempters, SA) according to the Columbia-Suicide Severity Rating Scale (C-SSRS) (20). A suicide attempt was defined as a self-damaging act carried out with certain intention to die, and was distinguished from self-mutilation, the use of substances or non-compliance with medical treatment (5). In the SA group, 34 patients were serious or violent SA. Violent suicide attempt was defined according to the criteria by Åsberg et al. (21) (i.e., hanging, drowning, jumping from heights, and suicide attempts with firearms or knives). Serious suicide attempt was defined according to the medical damage associated with the suicidal act (i.e., patient required hospitalization in intensive care) (22). Moreover, 63 SA were re-attempters (≥2 suicide attempt during their lifetime). Detailed characteristics of suicide attempts are described in Table 1.

**Table 1:** Characteristics of suicide attempts.

**Method of suicide attempt**	**Number of patients**
Medical intoxication (serious)	*N* = 81 (18)
Cutting	*N* = 7
Jumping	*N* = 5
Hanging	*N* = 3
Firearm	*N* = 1
Number of suicide attempts	Number of patients
1	*N* = 52
2	*N* = 24
3	*N* = 20
>4	*N* = 19
Age at first suicide attempt	Mean ± SEM = 28.41 ± 1.33

The study protocol was approved by the local research ethics committee (CPP Montpellier Sud-Méditerranée IV, CHU Montpellier) and was carried out according to the tenets of the Declaration of Helsinki. All participants signed a written informed consent.

### Clinical Assessment

At admission, patients were interviewed to collect their demographic data: age, education level, number of children, professional situation, civil status, and smoking history. The French version of the Mini International Neuropsychiatric Interview (MINI 5.0) (23) was used by senior psychiatrists to assess current and lifetime Axis I psychiatric disorders. Depression symptomatology was evaluated with the MADRS (19), and manic symptomatology with the Young Mania Rating Scale (YMRS) (24). Current psychotropic medication and daily dosage were recorded to calculate a general index of medication burden. The dosage of each drug was coded from 0 to 4, as previously described (25). The total medication burden was calculated by summing all the individual drug codes for the same patient. Finally, history of childhood trauma was evaluated using the Childhood Trauma Questionnaire (CTQ) (26).

### Cognitive Tasks

After clinical assessment patients performed several cognitive tasks.

#### Verbal IQ

Verbal IQ was evaluated using the French version of the National Adult Reading Test (NART) (27). The test comprises 50 irregular words in French violating grapheme-phoneme rules. Patients have to read the words with the correct pronunciation.

#### Attention

Sustained attention was evaluated using the letter-cancellation task of the d2 test (28, 29). d2 test consists of several series of letters. The patient have to double mark as fast as possible “d” in the middle of distractors.

#### Complex Emotion Recognition

The French version of the RMET (13, 30) was used to assess emotion recognition. In this test, 36 photographs of the eyes that express different complex emotions are presented. Participants were asked to choose among four adjectives the one that best describes each picture (three foil adjectives and one correct adjective). If necessary, the definition of each adjective was provided. The score was the total number of correct answers. The 36 images were also classified in three valence categories based on Harkness et al. (14): 8 positive (e.g., friendly), 12 negative (e.g., upset), and 16 neutral (e.g., reflective).

### Data Analysis

For sociodemographic and clinical variables, qualitative/categorical variables (i.e., sex, civil status, professional activity, current psychiatric comorbidity, etc.) were compared with the Chi-square test and quantitative/continuous variables (i.e., MADRS score, YMRS score, NART score, etc.) with the *t-*test. Spearman correlations were used to identify the variables (depression, mania, medication burden, verbal intelligence, and attention) associated with the RMET scores.

Then, mixed logistic models were performed, using as dependent variable the correct/incorrect identification of each RMET image (Right = 1/Wrong = 0). Suicide status and adjusting covariates (sex, lifetime bipolar disorder, verbal IQ using the NART total score, and attention using the GZ-F score from the d2 test) were used as fixed effects. The patient random effects and time random slopes were added to take into account the intra-patient correlation structure. This analysis was performed for each RMET valence category (positive, negative and neutral) and for the total RMET score, separately. The likelihood ratio test and Wald test were used to test significance.

The alpha significance level was fixed at 0.05. All statistical analyses were performed using R 3.5.3.

## Results

### Sample Characteristics and Correlations With the RMET Score

Compared with the AC group, current post-traumatic stress disorder (χ^2^ = 4.47, *p* < 0.035) and antipsychotic intake (χ^2^ = 6.87, *p* < 0.009) were more frequent, and suicidal ideation (item 10 of the MADRS) was higher (*t*_172_ = −3.73, *p* < 0.001) in the SA group. Similarly, the CTQ total score was higher (*t*_176_ = −2.73, *p* < 0.007) and history of physical abuse (χ^2^ = 4.37, *p* < 0.036) and of sexual abuse (χ^2^ = 4.19, *p* < 0.041) was more frequent in the SA than AC group. Sociodemographic data and other clinical variables, as well as verbal IQ and attention (d2) scores were comparable between groups (all *p* > 0.050; Table 2).

**Table 2:** Descriptive characteristics of the sample.

	**Lifetime SA**	**AC**	***p*-values**
*N* =	115	95	
**Sociodemographic data**			
Age, years	40.75 ± 1.20	41.80 ± 1.34	*p* < 0.559
Women, *n* (%)	83 (72.2%)	62 (65.3%)	*p* < 0.281
Years of education	13.72 ±0.29	14.01 ±0.27	*p* < 0.469
Sep./Div./Wid., *n* (%)	26 (23.0%)	21 (22.3%)	*p* < 0.909
Children, *n* (%)	64 (56.6%)	57 (60.6%)	*p* < 0.561
Professionally active/Student, *n* (%)	60 (53.1%)	45 (48.4%)	*p* < 0.501
Current smoker, *n* (%)	58 (51.8%)	38 (40.4%)	*p* < 0.251
**Clinical variables**			
Current anxiety disorder, *n* (%)	72 (68.6%)	52 (58.4%)	*p* < 0.143
Current eating disorder, *n* (%)	10 (8.9%)	3 (3.3%)	*p* < 0.103
Current alcohol abuse/dep., *n* (%)	17 (15.6%)	13 (14.3%)	*p* < 0.796
Current substance abuse/dep., *n* (%)	17 (15.3%)	9 (9.7%)	*p* < 0.229
Current PTSD, *n* (%)	18 (15.8%)	6 (6.4%)	*p* < 0.035
Current mixed episode, *n* (%)	9 (8.0%)	10 (10.5%)	*p* < 0.536
Lifetime bipolar disorder, *n* (%)	52 (45.2%)	40 (42.1%)	*p* < 0.651
Depressive symptomatology (MADRS)	28.14 ±0.92	26.18 ± 1.10	*p* < 0.173
Suicidal ideation (Item 10 MADRS)	3.17 ±0.20	2.10 ±0.20	*p* < 0.001
Mania symptomatology (YMRS)	1.71 ±0.42	1.53 ±0.35	*p* < 0.756
**Medication**			
Antidepressants, *n* (%)	72 (66.1%)	54 (65.9%)	*p* < 0.977
Anxiolytics, *n* (%)	75 (68.8%)	48 (58.5%)	*p* < 0.142
Antiepileptics, *n* (%)	28 (25.7%)	22 (26.8%)	*p* < 0.859
Antipsychotics, *n* (%)	58 (53.2%)	28 (34.1%)	*p* < 0.009
Lithium, *n* (%)	10 (9.2%)	10 (12.2%)	*p* < 0.500
Medication burden	3.82 ±0.22	3.54 ±0.26	*p* < 0.405
**Neuropsychological variables**			
Verbal IQ (NART)	20.53 ±0.51	20.98 ± 0.44	*p* < 0.512
Attention (d2)	349.15 ± 9.33	364.94 ± 10.12	*p* < 0.254
**Childhood Trauma Questionnaire (CTQ) Low/Severe/Moderate**			
Total	52.27 ± 1.99	44.73 ± 1.87	*p* < 0.007
Physical Abuse, *n* (%)	37 (38.5%)	21 (24.1%)	*p* < 0.036
Physical Neglect, *n* (%)	49 (51.0%)	36 (41.9%)	*p* < 0.215
Emotional Abuse, *n* (%)	69 (72.6%)	49 (59.0%)	*p* < 0.056
Emotional Neglect, *n* (%)	78 (80.4%)	59 (70.2%)	*p* < 0.112
Sexual Abuse, *n* (%)	40 (41.7%)	24 (27.3%)	*p* < 0.041

*SA, Suicide attempters; AC, Affective controls; Sep./Div./Wid., Separated/Divorced/Widowed; PTSD, Post-traumatic stress disorder; MADRS, Montgomery and Åsberg Depression Rating Scale; YMRS, Young Mania Rating Scale; IQ, Intelligence quotient; NART, National verbal learning task*.

The RMET scores were positively correlated with verbal IQ and attention (d2) scores, and education level (Table 3).

**Table 3:** Spearman correlations between Reading the Mind in the Eyes Test scores and sociodemographic, clinical and cognitive variables.

	**RMET Positive**	**RMET Negative**	**RMET Neutral**	**RMET Total**
Age	*r* = −0.095	*r* = −0.104	*r* = −0.061	*r* = −0.120
Years of education	***r*** **=** 0.157^*^	*r* = 0.075	***r*** **=** 0.314^***^	***r*** **=** 0.268^**^
Depressive symptomatology (MADRS)	*r* = 0.091	*r* = 0.064	*r* = 0.029	*r* = 0.071
Suicidal ideation (Item 10 MADRS)	*r* = 0.015	*r* = 0.146	*r* = 0.044	*r* = 0.094
Mania symptomatology (YMRS)	*r* = −0.016	*r* = −0.047	*r* = −0.103	*r* = −0.067
Medication burden	*r* = 0.006	*r* = 0.127	*r* = 0.076	*r* = 0.092
Days between last SA and evaluation	*r* = −0.001	*r* = −0.025	*r* = −0.101	*r* = −0.090
Verbal IQ (NART)	***r*** **=** 0.233^***^	*r* = 0.118	***r*** **=** 0.243^***^	***r*** **=** 0.275^***^
Attention (d2)	***r*** **=** 0.297^***^	***r*** **=** 0.136^*^	***r*** **=** 0.165^*^	***r*** **=** 0.272^***^
CTQ total score	*r* = 0.010	*r* = 0.038	*r* = −0.015	*r* = 0.017

* *p < 0.05*;

** *p < 0.01*;

*** *p < 0.001*.

*RMET, Reading the Mind in the Eyes Test; MADRS, Montgomery and Åsberg Depression Rating Scale; YMRS, Young Mania Rating Scale; SA, Suicide attempt; IQ, Intelligence quotient; NART, National verbal learning task; CTQ, Childhood Trauma Questionnaire*.

### RMET Score and History of Suicide Attempt(s)

**Figure 1:**
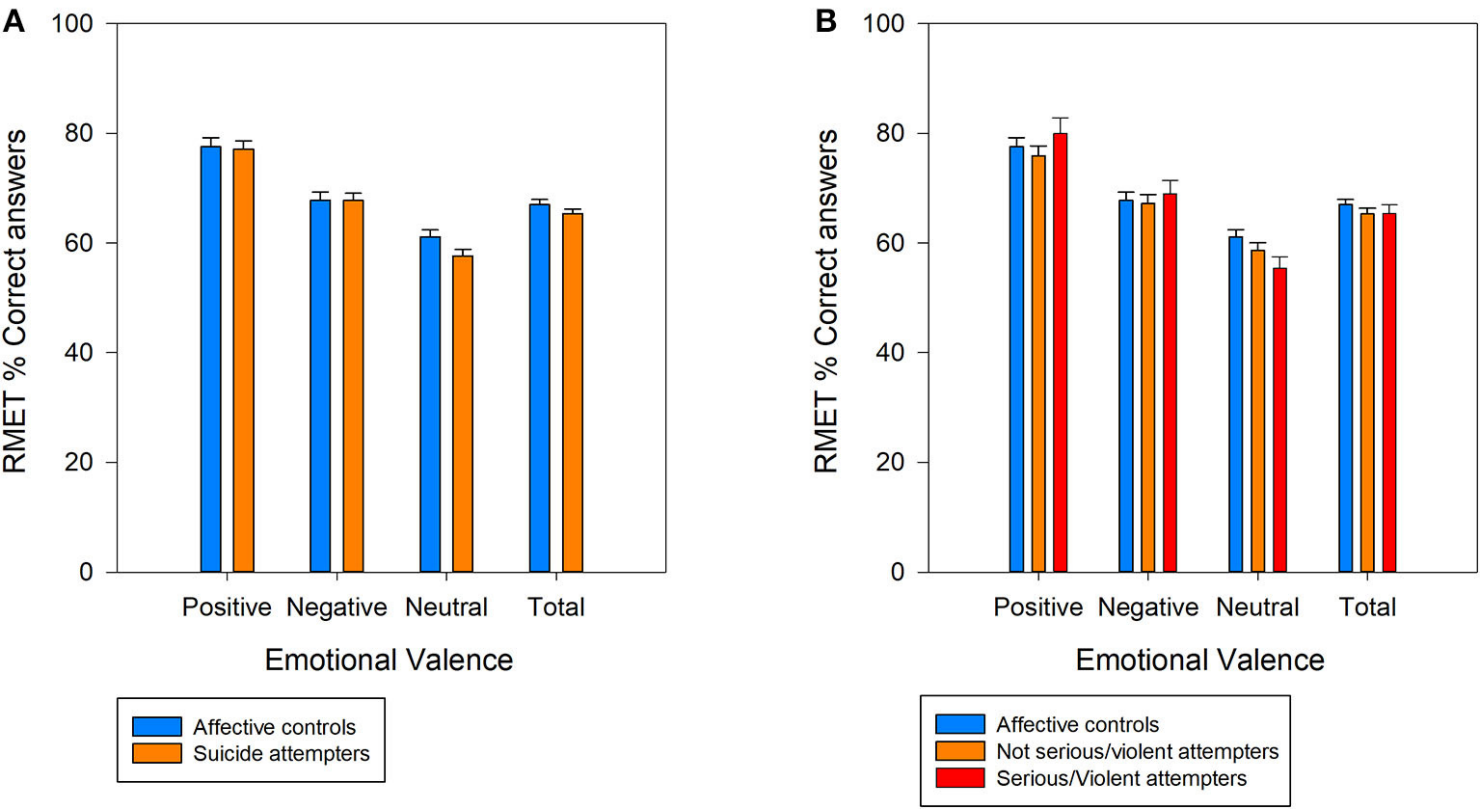
Percentage (mean ± SEM) of correct answers for all photographs (total) and for each of three valence categories (positive, negative, and neutral) of the Reading the Mind in the Eyes test; **(A)** Comparison between patients with current major depressive episode (MDE) with and without (affective controls) lifetime suicide attempt history; **(B)** Comparison of patients with MDE without (affective controls) and with history of serious/violent suicide attempts, or history of non-serious/ non-violent suicide attempts.

First, computing the random effects for all tested variables showed that time random slopes did not significantly improve the model (all *p* > 0.050). Therefore, all models were computed using only the patient random intercept. Figure 1 shows Mean ± SEM of the percentages. Comparison of the total RMET score and the scores for each valence category showed that only the RMET score for the neutral valence category was lower in the SA than AC group (OR = 0.87, 95% CI [0.75, 0.99]) (Figure 1A). Conversely, there was not significant difference between groups for the total RMET score and also for the positive and negative valence categories (all *p* > 0.050).

When SA were classified in patients with and without history of violent/serious suicide attempt, the RMET score for the neutral valence category showed a trend for the group factor (χ^2^ = 5.63, *p* < 0.059). Indeed, this score tended to be lower in the violent/serious SA group compared with the AC group (OR = 0.79, 95% CI [0.64, 0.96]), but not with the non-violent/serious SA group (Figure 1B). The positive and negative valence category scores and the total RMET score were not different among groups (all *p* > 0.050).

Comparison of the RMET scores in suicide re-attempters, in patients who attempted suicide only once, and in the AC group did not highlight any significant difference (all *p* > 0.050).

All the results for the fixed effects tested, including covariates, are shown in Supplementary Materials.

## Discussion

Our results showed that the interpretation of social complex emotions is impaired in depressed patients with history of suicide attempt. Suicide attempters performed worse than patients with depression without suicide history when interpreting neutral emotions. Our results are in agreement with studies reporting that suicide attempters have difficulties in the interpretation of facial emotions (7), and show alterations in brain activation during facial emotional processing (8, 12, 31). However, when considering the RMET scores, previous studies failed to show differences between patients with depression and history or not of suicide attempts (16, 18). Several reasons may explain these discrepancies. First, Szanto et al. (18) used a sample of elderly patients, and normal aging has been associated with worse emotional recognition (32). Moreover, they did not adjust for attention and verbal intelligence. Although the RMET does not require high executive demands (13), its performance is closely related to the verbal IQ score (33). Our detection of differences between SA and AC might be explained by the fact that we adjusted our analysis for these variables. Interestingly, our results showed that the RMET score for the neutral valence category tended to be worse in serious/violent suicide attempters. Similarly, Szanto et al. (18) found a negative correlation between suicide severity and total RMET score.

Conversely to our initial hypothesis, interpretation of negative emotions was not impaired in suicide attempters, only that of neutral emotions. Ai et al. (31) reported altered functions in the fusiform gyrus in suicide attempters compared with non-attempters during emotional processing of all kinds of emotional faces, including neutral faces. Fusiform gyrus is a key brain region in facial recognition, and systematically involved in prosopagnosia (34). During emotional faces recognition it has been shown that fusiform gyrus was highly interconnected with the orbitofrontal cortex and the amygdala (35), both areas impaired during emotional processing in suicidal patients (8, 12). Therefore, a possible mechanism to explain impaired emotion recognition in suicide attempters may be a disrupted connectivity between fusiform gyrus, prefrontal and limbic areas. Neutral expressions are inherently ambiguous, thus facilitating an overinterpretation of their valence (36). Maniglio et al. (37) showed that in the general population, people with more depressive symptomatology, death wishes and suicidal ideation and planning have difficulties in recognizing neutral facial expressions.

Our study has some limitations. Its cross-sectional nature limits causality inference; future studies should use a prospective design. Moreover, patients with psychosis who are characterized by impaired emotional recognition (38) were not included, thus preventing the result generalization to all patients with suicidal risk. Finally, social functioning was not assessed, although it may provide insights into how emotion recognition impairment may affect the social daily life of suicidal patients.

In conclusion, our results show that recognition of neutral emotions is impaired in patients with depression and history of suicide attempt(s), particularly those with history of violent/serious suicide attempt. The development of programs to better identify and interpret neutral emotions may be considered to prevent suicidal risk in patients with depression.

## Data Availability Statement

The original contributions presented in the study are included in the article/Supplementary Materials, further inquiries can be directed to the corresponding author/s.

## Ethics Statement

The studies involving human participants were reviewed and approved by CPP Montpellier Sud-Méditerranée IV, CHU Montpellier. The patients/participants provided their written informed consent to participate in this study.

## Author Contributions

Recruiting participants: CG, MS, PC, and EO. Performed the psychiatric interviews: PC and EO. Performed the neuropsychological assessment: CG and MS. Reviewed the specific literature: IF, AA-C, AS, and EO. Formulated the problem and hypothesis: IF, AA-C, AS, PC, and EO. Analyzed data: AA-C and JD. Wrote the paper: IF, AA-C, CG, and JD. Approved the final version of paper: IF, AA-C, AS, CG, JD, MS, PC, and EO. All authors contributed to the article and approved the submitted version.

## Conflict of Interest

The authors declare that the research was conducted in the absence of any commercial or financial relationships that could be construed as a potential conflict of interest.
