# Supplementary material for: I Cannot Read Your Eye Expression: Suicide Attempters Have Difficulties in Interpreting Complex Social Emotions
Source: Front Psychiatry. 2020 Nov 10;11:543889. doi: 10.3389/fpsyt.2020.543889 (PMC7683427; doi:10.3389/fpsyt.2020.543889)
Supplement: Supplementary file 1 [file Data_Sheet_1.docx]

**Supplementary material Table 1: Fixed effects from the mixed logistic regression analysis when evaluating the effect of history of lifetime suicide attempt:**

| RMET Positive |  |  |  |  | RMET Negative |  |  |  |  |
| --- | --- | --- | --- | --- | --- | --- | --- | --- | --- |
|  | χ^2^ | *p-value* | B *(SE)* | OR [95% CI] |  | χ^2^ | *p-value* | B *(SE)* | OR [95% CI] |
| **Sex (W=1)** | **7.15** | **.008** | **.36 (.14)** | **1.44 [1.10, 1.88]** | Sex (W=1) | .70 | .401 | .08 (.09) | 1.08 [.89, 1.31] |
| Lifetime bipolar disorder (Y=1) | .32 | .573 | .07 (.13) | 1.08 [.88, 1.39] | Lifetime bipolar disorder (Y=1) | .19 | .666 | - .04 (.09) | .96 [.81, 1.14] |
| **Verbal IQ** | **8.62** | **.003** | **.04 (.01)** | **1.04 [1.01, 1.06]** | Verbal IQ | .57 | .450 | .01 (.01) | 1.01 [.99, 1.03] |
| **Attention** | **9.68** | **.002** | **.01 (.01)** | **1.00 [1.00, 1.01]** | **Attention** | **4.35** | **.037** | **.01 (.01)** | **1.00 [1.00, 1.01]** |
| Lifetime Suicide Attempt (Y=1) | .01 | .904 | - .02 (.13) | .98 [.76, 1.27] | Lifetime Suicide Attempt (Y=1) | .01 | .956 | - .01 (.09) | .99 [.83, 1.19] |
| RMET Neutral |  |  |  |  | RMET Total |  |  |  |  |
|  | χ^2^ | *p-value* | B *(SE)* | OR [95% CI] |  | χ^2^ | *p-value* | B *(SE)* | OR [95% CI] |
| Sex (W=1) | .53 | .467 | - .06 (.08) | .94 [.81, 1.10] | Sex (W=1) | 1.04 | .308 | .06 (.06) | 1.06 [.94, 1.19] |
| **Lifetime bipolar disorder (Y=1)** | **9.36** | **.002** | **- .22 (.07)** | **.80 [.69, .92]** | **Lifetime bipolar disorder (Y=1)** | **3.49** | **.061** | **- .11 (.06)** | **.89 [.81, 1.01]** |
| **Verbal IQ** | **11.41** | **.001** | **.03 (.01)** | **1.03 [1.01, 1.04]** | **Verbal IQ** | **13.64** | **.001** | **.02 (.01)** | **1.02 [1.01, 1.03]** |
| Attention | 2.99 | .084 | .01 (.01) | 1.00 [.99, 1.00] | **Attention** | **11.71** | **.001** | **.01 (.01)** | **1.00 [1.00, 1.01]** |
| **Lifetime Suicide Attempt (Y=1)** | **4.03** | **.044** | **- .14 (.07)** | **.87 [.75, .99]** | Lifetime Suicide Attempt (Y=1) | 1.72 | .189 | - .07 (.06) | .93 [.83, 1.04] |

**Supplementary material Table 2: Fixed effects from the mixed logistic regression analysis when evaluating the effect of history of violent/serious suicide attempt:**

| RMET Positive |  |  |  |  | RMET Negative |  |  |  |  |
| --- | --- | --- | --- | --- | --- | --- | --- | --- | --- |
|  | χ^2^ | *p-value* | B *(SE)* | OR [95% CI] |  | χ^2^ | *p-value* | B *(SE)* | OR [95% CI] |
| **Sex (W=1)** | **7.61** | **.006** | **.38 (.14)** | **1.47 [1.02, 1.91]** | Sex (W=1) | .81 | .401 | .09 (.09) | 1.09 [.90, 1.32] |
| Lifetime bipolar disorder (Y=1) | .14 | .704 | .05 (.13) | 1.05 [.81, 1.36] | Lifetime bipolar disorder (Y=1) | .26 | .666 | - .05 (.09) | .95 [.79, 1.14] |
| **Verbal IQ** | **7.68** | **.006** | **.04 (.01)** | **1.04 [1.01, 1.07]** | Verbal IQ | .48 | .450 | .01 (.01) | 1.01 [.99, 1.03] |
| **Attention** | **10.35** | **.001** | **.01 (.01)** | **1.00 [1.00, 1.01]** | **Attention** | **4.53** | **.037** | **.01 (.01)** | **1.00 [1.00, 1.01]** |
| Violent/Serious suicide attempt | .44 | .441 |  |  | Violent/Serious suicide attempt | .38 | .826 |  |  |
| No (Y=1) |  |  | - .09 (.14) | .92 [.69, 1.21] | No (Y=1) |  |  | - .03 (.09) | .97 [.80, 1.18] |
| Yes (Y=2) |  |  | .16 (.19) | 1.18 [.81, 1.72] | Yes (Y=2) |  |  | .05 (.13) | 1.06 [.82, 1.36] |
| RMET Neutral |  |  |  |  | RMET Total |  |  |  |  |
|  | χ^2^ | *p-value* | B *(SE)* | OR [95% CI] |  | χ^2^ | *p-value* | B *(SE)* | OR [95% CI] |
| Sex (W=1) | .73 | .393 | - .07 (.08) | .94 [.80, 1.09] | Sex (W=1) | 1.03 | .310 | .06 (.06) | 1.06 [.94, 1.19] |
| **Lifetime bipolar disorder (Y=1)** | **8.11** | **.004** | **- .21 (.07)** | **.81 [.70, .94]** | **Lifetime bipolar disorder (Y=1)** | **3.42** | **.065** | **- .11 (.06)** | **.89 [.80, 1.01]** |
| **Verbal IQ** | **12.00** | **.001** | **.03 (.01)** | **1.03 [1.01, 1.04]** | **Verbal IQ** | **12.55** | **.001** | **.02 (.01)** | **1.02 [1.01, 1.03]** |
| Attention | 2.54 | .111 | .01 (.01) | 1.00 [.99, 1.00] | **Attention** | **11.71** | **.001** | **.01 (.01)** | **1.00 [1.00, 1.01]** |
| **Violent/Serious suicide attempt** | **5.63** | **.059** |  |  | Violent/Serious suicide attempt | 1.72 | .444 |  |  |
| No (Y=1) |  |  | - .10 (.08) | .90 [.77, 1.05] | No (Y=1) |  |  | - .07 (.06) | .93 [.82, 1.04] |
| Yes (Y=2) |  |  | - **.24 (.10)** | **.79 [.64, .96]** | Yes (Y=2) |  |  | - .07 (.08) | .93 [.79, 1.09] |

**Supplementary material Table 3: Fixed effects from the mixed logistic regression analysis when evaluating the effect of repeated suicide attempts:**

| RMET Positive |  |  |  |  | RMET Negative |  |  |  |  |
| --- | --- | --- | --- | --- | --- | --- | --- | --- | --- |
|  | χ^2^ | *p-value* | B *(SE)* | OR [95% CI] |  | χ^2^ | *p-value* | B *(SE)* | OR [95% CI] |
| **Sex (W=1)** | **6.93** | **.008** | **.36 (.14)** | **1.44 [1.10, 1.88]** | Sex (W=1) | .71 | .401 | .08 (.09) | 1.08 [.89, 1.31] |
| Lifetime bipolar disorder (Y=1) | .32 | .571 | .07 (.13) | 1.08 [.83, 1.39] | Lifetime bipolar disorder (Y=1) | .19 | .662 | - .04 (.09) | .96 [.80, 1.14] |
| **Verbal IQ** | **8.43** | **.004** | **.04 (.01)** | **1.04 [1.01, 1.07]** | Verbal IQ | .57 | .451 | .01 (.01) | 1.01 [.99, 1.03] |
| **Attention** | **9.52** | **.002** | **.01 (.01)** | **1.00 [1.00, 1.01]** | **Attention** | **4.32** | **.038** | **.01 (.01)** | **1.00 [1.00, 1.01]** |
| Suicide attempts ≥ 2 | .02 | .989 |  |  | Suicide attempts ≥ 2 | .03 | .984 |  |  |
| No (Y=1) |  |  | - .01 (.16) | .99 [.72, 1.36] | No (Y=1) |  |  | - .02 (.11) | .98 [.79, 1.22] |
| Yes (Y=2) |  |  | - .02 (.15) | .98 [.73, 1.32] | Yes (Y=2) |  |  | .01 (.11) | 1.01 [.82, 1.23] |
| RMET Neutral |  |  |  |  | RMET Total |  |  |  |  |
|  | χ^2^ | *p-value* | B *(SE)* | OR [95% CI] |  | χ^2^ | *p-value* | B *(SE)* | OR [95% CI] |
| Sex (W=1) | .46 | .464 | - .06 (.08) | .94 [.81, 1.09] | Sex (W=1) | 1.03 | .310 | .06 (.06) | 1.06 [.94, 1.19] |
| **Lifetime bipolar disorder (Y=1)** | **9.13** | **.003** | **- .22 (.07)** | **.80 [.69, .93]** | **Lifetime bipolar disorder (Y=1)** | **3.44** | **.063** | **- .11 (.06)** | **.89 [.81, 1.01]** |
| **Verbal IQ** | **11.24** | **.001** | **.03 (.01)** | **1.03 [1.01, 1.04]** | **Verbal IQ** | **12.69** | **.001** | **.02 (.01)** | **1.02 [1.01, 1.03]** |
| Attention | 2.94 | .086 | .01 (.01) | 1.00 [.99, 1.00] | **Attention** | **10.89** | **.001** | **.01 (.01)** | **1.00 [1.00, 1.01]** |
| Suicide attempts ≥ 2 | 4.13 | .126 |  |  | Suicide attempts ≥ 2 | 1.74 | .419 |  |  |
| No (Y=1) |  |  | - .13 (.09) | .88 [.74, 1.05] | No (Y=1) |  |  | - .07 (.07) | .93 [.82, 1.07] |
| Yes (Y=2) |  |  | - .16 (.08) | .85 [.73, 1.01] | Yes (Y=2) |  |  | - .08 (.07) | .92 [.81, 1.05] |
